# International collaboration for the development of clinical guidelines in low and middle-income countries: case study on the development of a national framework and clinical guidelines for diabetic retinopathy in Ghana

**DOI:** 10.1038/s41433-022-02002-9

**Published:** 2022-05-19

**Authors:** Nyawira Mwangi, Kwesi Nyan Amissah-Arthur, Imoro Zeba Braimah, Osei Sarfo-Kantanka, Josephine Akpalu, Bridgid Akrofi, Samuel Bert Boadi-Kusi, Yacoba Atiase, Ernest Yorke, Michael Gichangi, Hannah Faal, James Addy

**Affiliations:** 1 Kenya Medical Training College, Nairobi, Kenya; 2 London School of Hygiene & Tropical Medicine, London, UK; 3 Ophthalmology Unit, Department of Surgery, Korle Bu Teaching Hospital, College of Health Sciences, School of Medicine and Dentistry, University of Ghana, Accra, Ghana; 4 Department of Medicine, Kwame Nkrumah University of Science and Technology, Kumasi, Ghana; 5 Department of Medicine and Therapeutics, University of Ghana School of Medicine and Dentistry, Accra, Ghana; 6 National Diabetes Centre, Korle Bu Teaching Hospital, Accra, Ghana; 7 Department of Optometry and Vision Science, School of Allied Health Sciences, University of Cape Coast, Cape Coast, Ghana; 8 Ophthalmic Services Unit, Ministry of Health, Nairobi, Kenya; 9 University of Calabar Teaching Hospital, Calabar, Nigeria; 10 Eye Care Unit, Ghana Health Service, Accra, Ghana

## Abstract

**Background:**

Diabetic retinopathy is a leading cause of blindness in many countries across the world. Ghana has seen a rise in diabetic retinopathy and is working on various strategies to prevent blindness. Clinical guidelines are seen as a promising strategy for improving quality and reducing cost of care. Little is known about the processes of collaborative guideline development in the African context.

**Methods:**

This case study discusses the process of developing clinical guidelines for diabetic retinopathy in Ghana via a collaboration with the Kenya team that had previously developed guidelines for Kenya.

**Results:**

The main lesson learnt was the ability to overcome challenges. The main output achieved was the draft national framework, guidelines and training slides on the guidelines.

**Conclusion:**

Horizontal international collaboration can aid development of clinical guidelines.

## Introduction

The burden of diabetes and diabetic retinopathy (DR) in Africa is projected to increase due to changes in demography, urbanisation and lifestyle. Clinical guidelines are important in health system strengthening, but developing such guidelines is complex [1]. Strategic collaboration between national and international stakeholders may confer benefits to the process of developing clinical guidelines.

The Ghana–Kenya collaboration is an example of a horizontal collaboration between two African countries facilitated by the Diabetic Retinopathy Network (DR-NET) (thus a triangular collaboration) to improve capacity for DR care. The role of intercountry collaboration in guideline development in low and middle-income countries (LMICs) has not been documented [2, 3]. To our knowledge, this is the first example of collaboration between two LMICs to develop clinical guidelines for eye care.

## Case description

In 2017 Kenya launched the national DR guidelines in electronic and print formats, point of care materials, as well as tools for quality assurance, training, monitoring and evaluation. Documenting this experience can inform the development of guidelines in similar contexts [1, 4].

**Fig. 1:**
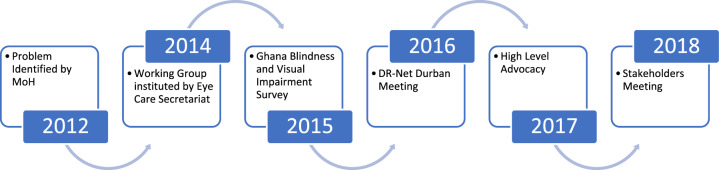
A summary of the process of collaborative guideline development from 2012 to 2018. The process targeted multiple stakeholders and strategies.

The need for DR guideline in Ghana was recognised in 2012 and a working group instituted in 2014 (Fig. 1). In 2017 the formulation of a national framework and DR guidelines commenced, and in 2018 the Kenya team was invited to share learning at a stakeholders meeting. These stakeholders collectively became the guideline developers, representing policy, multidisciplinary clinical practice (we included all relevant professional groups in Ghana), academia, programme implementers (such as non-governmental organisations), private sector and researchers.

The IQ scientific centre for quality of health care, in their tool for international collaboration in guideline development, has identified six important steps in the development of international collaboration for guideline development [5] (Table 1). We did not develop a protocol for the collaboration *apriori*, but we documented the process of developing the collaboration for transparency and future reference. We describe how our process related to these steps below.

**Table 1:** Steps for international collaboration in guideline development.

Step-by-step plan for international collaboration
i. Step 1: Get to know each other, create a foundation of trust and set goals for collaboration
ii. Step 2: Analyse the work methods, compare the delivered products and discuss possible differences
iii. Step 3: Determine which components the development groups wish to collaborate on and come to a documented agreement, including agreements on possible differences
iv. Step 4: Draw up a plan of approach based on the agreement, use a uniform format and establish communication channels
v. Step 5: Make agreements in this plan of approach on how the evaluation of each other’s products and insight differences will be handled
vi. Step 6: Implement the plan of approach and make arrangements about the products’ revision procedure

### Step 1: Get to know each other, create a foundation of trust and set goals for collaboration

Prior to the collaboration, DR-NET international workshops provided an opportunity for the Ghana and Kenya teams to meet and develop a shared vision for developing the guidelines. This early interaction was useful because developing trust (which is a prerequisite for collaboration) requires time and personal contact. Various research on collaboration has documented the importance of corporate and personal trust [6–8]. We did not measure trust, but the willingness to collaborate was evidence of trust. Although long-term relationships between north and south partners via LINKS programmes has attracted wide attention, there has been little documentation of such south-south partnerships [9].

### Step 2: Analyse the work methods, compare the delivered products and discuss possible differences

In this step we sought to clarify: (1) The aspiration of the collaboration, which was the development of a national DR framework and draft DR guidelines for Ghana (2) The experiences, lessons learnt and outputs from the Kenya model, and also the similarities/nuances to the Ghanaian context. (3) The specific collaboration and guideline development activities that would be useful, such as in-country workshops (4) We also discussed whether we could just adopt the Kenyan guidelines, or develop Ghanaian guidelines de novo. For pragmatic reasons, and for synergy, we eventually achieved consensus to balance between the two options, and learn from the Kenya experience. This formal and informal dialogue on whether it was right for Ghana to adopt the Kenya guidelines was very useful. As can be expected, there were stakeholders who strongly favoured each of the options. The best practice in collaboration is to allow expression and debate over these options, rather than use suppression [8, 10]. We did not have overt conflict, but this is one example of potential conflict. Similar conflicts have also been reported in guideline development [1]. Managing conflict is an essential skill for both collaboration and guideline development.

### Step 3: Determine which components the development groups wish to collaborate on

We brainstormed on the priorities for collaboration. Prioritisation was based on the areas that the Ghanaian team considered very crucial. The five main areas for collaboration were: sharing relevant evidence, engaging stakeholders, identifying key guiding principles for Ghanaian guidelines, drafting the guidelines, and documenting the process. We then outlined a programme for how these priorities would be achieved.

### Step 4: Draw up a plan of approach and establish communication channels

We used email, official letters and face to face discussions for communication. The stakeholder meeting in Accra, Ghana was attended by the stakeholders from Ghana as well as representatives from Kenya,DR-NET and Nigeria. This provided a window of opportunity to further share learning among three African countries.

To facilitate broad input in the guideline development, representatives from nine disciplines (non-communicable diseases, endocrinology, general medicine, public health, ophthalmology, optometry, pharmacy and nursing) were invited to participate. Primary, secondary and tertiary level facilities were represented, as were universities and non-governmental organisations. This diversity helped to include different viewpoints, and to facilitate buy-in and ownership. We would recommend that guideline developers similarly aim to span across all these professional boundaries for successful guideline development. Bringing together so many different experts was quite time-consuming, as has been noted in other collaborations [7]. However, it is also an important component of a successful collaboration [4, 6]. All these participants contributed their time and expertise on a voluntary basis, an important input described in other partnerships [9].

### Step 5: Make agreements in this plan of approach on how evaluation and differences will be handled

We strongly encouraged country ownership of the process and outputs. It is important to involve decision-makers that have the authority and credibility to support a guideline’s claims to be a national (or regional) scope. The fit within the Ghana’s Ministry of Health agenda facilitated buy-in in the broader health sector. Ownership of the process by end-users such as health professionals who are the guideline implementers is also important [4]. It was especially pertinent to involve team leads from non-communicable diseases, endocrinology and ophthalmology, as well as professional associations.

Further, it was necessary to specify the roles and tasks of different stakeholders. For example, the administrative tasks of coordinating activities, group communication, reporting and funding were performed by the DR-NET and the Ghana team. While role clarity was essential, we were also comfortable with some overlap or blurring of roles. This helped to maintain efficiency, continuity of the process, goodwill of stakeholders and the credibility of the process. Katisi et al. have noted that antagonism and role boundary conflicts may occur when there is overlap of roles, but we did not experience this [10]. This is likely because the participants were experienced in functioning within collaboration, and the Ghanaian team provided good stewardship.

### Step 6: Implement the plan of approach

In guideline development it is important to obtain ‘bottom up’ views on the best ways to tackle complex health care problems such as DR. Stakeholders must be given a real opportunity to discuss the issues and the evidence, and to reach consensus [7]. In our case, this was achieved through the stakeholder workshop. We provided stakeholders with some well-written reading materials ahead of the workshop and details of the collaboration were presented to stakeholders at the start of the workshop. As a result, the partnership was perceived as important, and prompted the stakeholders to engage with each other [7]. Initially we were rather concerned about putting so many people in one room as we thought it might be a threat to maintaining focus, however, it ended up being very productive due to effective engagement.

We had skilled facilitation for the four-day workshop, which was sufficient to discuss the evidence and develop the recommendations for Ghana. We presented collated evidence to stakeholders which they discussed in groups. We then used a modified nominal group technique to facilitate discussion of recommendations and consensus. This approach might usefully be replicated in other settings, provided the lead workshop facilitator has wide experience, expertise and credibility. Our lead facilitator was a boundary-spanner, bringing in technical expertise, experience with eye care management in different countries, familiarity of context and working with governments and non-government stakeholders. A guideline writing committee was formed after the workshop.

Eight distinct yet integrated foundational principles underpinning the Ghanaian DR guidelines were identified (Table 2). These principles reflect the values and priorities of the DR programme.

**Table 2:** Guiding principles for Ghanaian DR guidelines.

Guiding principle	How the principle is operationalised in the guidelines
Patient-centred focus	Recommendations should be focused on the health needs of the patient as they navigate the care pathway. Patient-centredness will be as valued as the clinical outcomes and markers of patient satisfaction identified.
Annual screening	All patients with diabetes should receive at least an annual DR screening exam (or more frequently in specific circumstances).
Screening at point of diabetes care	Photography-based screening will be provided at points of diabetes care.
Regional focus	Integrated patient care will be provided within the existing regionalised health care system, in recognition of regional peculiarities.
Holistic care	Health workers will continuously assess the needs of patients (even beyond eye care needs) and facilitate provision care to meet those needs, including preventive, promotive, curative, rehabilitative care and referral as appropriate. DR care will be integrated into diabetes care, which is in turn integrated into the NCDs strategy.
Bi-directional feedback	Point of care tools to enhance communication between care providers will include consultation and referral tools. These tools will include a section on feedback to the referring provider and to the patient.
Sustainability	The guidelines include strategies for capacity-development for implementation of the guidelines, such as training, supervision, integration in the health system and acquisition of the required infrastructure, technology and equipment.
Collaboration and partnership	The guidelines embrace international, national, regional, inter-institutional, inter-professional and public-private partnerships for the common goal of improvement in the quality of care.

Reflecting on the drivers for success, we found that effective communication, willingness to contribute and share expertise, transparency, flexibility, sustained commitment, and patience were all very important. The capacity to co-ordinate the collaboration and process of guideline development was also a core requirement. Shared leadership for this coordination role was demonstrated. For instance, the DR-NET provided coordination and finances for travel and workshops. The Ghana team organised all the in-country activities. A skilled external facilitator for the stakeholder workshop worked with all the teams to ensure that stakeholders were actively engaged.

The benefits of participating in this process were considered to be broader than just creating the DR framework and guidelines. For example, it enhanced the understanding of principles such as patient-centred care, which are transferable to other areas of health care. It also strengthened networks between participants, especially at country level. Such social capital is considered to be an important value of health partnerships that should be measured [8, 9]. The main outputs of the process were the draft national framework, guidelines and training slides on the guidelines. A summary of the documents has been shared with the Minister of Health and the Ghana Health Service. Training sessions have already been provided for the Ophthalmological Society of Ghana and the Ghana College of Physicians and Surgeons.

An important success factor was the ability to overcome challenges. Guideline development requires time, which was challenging given that this was not a dedicated guideline development group and all the team members had other professional responsibilities [1]. We found advance sharing of background documents and compiling discussion materials to be helpful for expediting review by members. There was considerable uncertainty about how the writing of the actual guideline document would be managed, because it is labour-intensive. A writing group was appointed, and we recommend this as an essential step. What has worked in Kenya is to have a highly skilled and self-driven person at the national level to dedicate a set period of time to putting together this document. However, this is may not always be a low-hanging fruit, given the health workforce challenges and competing tasks.

Some of the members were initially unclear on how to actively engage with the process, not being fully familiar with the processes of guideline development. This is not unusual given the relatively short history of guideline development within LMICs. The governance team helped to demystify some of policy, clinical or guideline development concepts, thus effectively connecting the various stakeholders to the process. This ability to communicate in the language of policymakers, clinicians, patients and guideline developers is important for collaborations for guideline development. It is an important knowledge-brokering task that helps stakeholders to add their voice to the process. Although we involved the Ghanaian Diabetes Association (a patients’ body) in the guideline development, engaging patient representatives and the general public in the process remained challenging. This is an important area for further interrogation.

Guideline development is an expensive process. The DR-NET provided the funding. Clinicians and academics provided in kind contributions of time, networks, skills and knowledge. All these are important costs [7]. In the short-term period further costs can be anticipated, such as for guideline production, dissemination, and training of implementers. In the long term, there will be costs for additional infrastructure, monitoring and evaluation. Provided the next steps in the guideline process are maintained, we can expect that over the next few years, there will be evidence of the cost-effectiveness of the guidelines, as well as data supporting the improvement in quality of care. Patients requiring DR services will be identified early and timely treatment will lead to cost-savings in the health system. These expectations necessitate collaboration and advocacy to mobilise resources.

### Reflections

The aim of this case description was two-fold. Firstly, to reflect on the pathway for the collaboration for the development of Ghana’s national framework and guidelines for DR. Secondly, to highlight the lessons learnt from this collaboration. An important strength of the paper is that we have examined the process of developing the collaboration, which is lacking in the literature. The main limitation is that this is a single case, which limits generalisability. The case is reported by those who were directly involved in its implementation, which may introduce bias by social desirability. However, we have triangulated perspectives from the different partners.

The collaboration increased the flow of information, resources, expertise and knowledge between the countries. An important product of the collaboration which was essential for its own effectiveness was synergy - the degree to which a partnership combined the strengths, perspectives, values and resources of all the partners. This may have reduced the lead time from the initiation to the completion of the process.

The DR-NET played important roles in the collaboration. First, in providing an opportunity for intercountry interaction. The literature on partnership recognises the need and importance of this role, which is commonly referred to as ‘boundary-spanning’. This helps to establish a climate of trust. Second, given the existing outfit of the DR-NET, we did not require extensive procedural elements for the collaboration, such as memorandums of understanding, which usually increase transactional costs [7, 8]. Third, the DR-NET participated in knowledge-sharing based on the experiences of collaboration in other ventures. Fourth, the DR-NET provided the financial resources for the activities as previously described.

## Conclusion

Documenting this case report contributes to the knowledge base on the functioning of partnerships for guideline development. It contributes to the global debate on the role of collaboration for health, and may inform future governance decisions within the DR-NET and other LINKS programmes. Future research might explore the cost-effectiveness, sustainability and role of such collaboration in other joint ventures, such as research.
